# Determination of the Proteolytic Cleavage Sites of the Amyloid Precursor-Like Protein 2 by the Proteases ADAM10, BACE1 and γ-Secretase

**DOI:** 10.1371/journal.pone.0021337

**Published:** 2011-06-17

**Authors:** Sebastian Hogl, Peer-Hendrik Kuhn, Alessio Colombo, Stefan F. Lichtenthaler

**Affiliations:** 1 German Center for Neurodegenerative Diseases (DZNE), Munich, Germany; 2 Adolf-Butenandt-Institute, Biochemistry, Ludwig-Maximilians-University, Munich, Germany; Thomas Jefferson University, United States of America

## Abstract

Regulated intramembrane proteolysis of the amyloid precursor protein (APP) by the protease activities α-, β- and γ-secretase controls the generation of the neurotoxic amyloid β peptide. APLP2, the amyloid precursor-like protein 2, is a homolog of APP, which shows functional overlap with APP, but lacks an amyloid β domain. Compared to APP, less is known about the proteolytic processing of APLP2, in particular in neurons, and the cleavage sites have not yet been determined. APLP2 is cleaved by the β-secretase BACE1 and additionally by an α-secretase activity. The two metalloproteases ADAM10 and ADAM17 have been suggested as candidate APLP2 α-secretases in cell lines. Here, we used RNA interference and found that ADAM10, but not ADAM17, is required for the constitutive α-secretase cleavage of APLP2 in HEK293 and SH-SY5Y cells. Likewise, in primary murine neurons knock-down of ADAM10 suppressed APLP2 α-secretase cleavage. Using mass spectrometry we determined the proteolytic cleavage sites in the APLP2 sequence. ADAM10 was found to cleave APLP2 after arginine 670, whereas BACE1 cleaves after leucine 659. Both cleavage sites are located in close proximity to the membrane. γ-secretase cleavage was found to occur at different peptide bonds between alanine 694 and valine 700, which is close to the N-terminus of the predicted APLP2 transmembrane domain. Determination of the APLP2 cleavage sites enables functional studies of the different APLP2 ectodomain fragments and the production of cleavage-site specific antibodies for APLP2, which may be used for biomarker development.

## Introduction

Regulated intramembrane proteolysis (RIP) occurs for an increasing number of membrane proteins, and is a molecular mechanism controlling the communication between cells [1], [2]. In the RIP process a membrane protein typically undergoes two consecutive proteolytic cleavages. The first one happens outside of the membrane and results in shedding of the ectodomain. The second cleavage, called intramembrane proteolysis, occurs within the transmembrane domain and leads to the secretion of a small peptide and the release of the intracellular domain into the cytosol. The proteolytic cleavage fragments act as versatile signaling molecules or are further degraded [3], [4]. RIP also occurs for the amyloid precursor protein (APP) family. This family comprises APP itself as well as its two homologs amyloid precursor-like proteins 1 and 2 (APLP1, APLP2) [5]. APP and APLP2 are ubiquitously expressed, whereas expression of APLP1 is restricted to the nervous system. The biological function of the APP protein family members is not yet fully understood. The three proteins are required for embryonic development, because in mice the triple knock-out of APP, APLP1 and APLP2 results in perinatal lethality, cranial abnormalities and cortical dysplasias [6]. In contrast, mice singly deficient in either APP, APLP1 or APLP2 alone as well as the double knock-out of APP and APLP1 do not show a phenotype or only a mild one [7], [8], [9], [10], suggesting functional overlap between the three family members. However, the double knock-out of APLP2 with either APP or APLP1 results in postnatal lethality [9], [10]. Together, these studies demonstrate that APLP2 is particularly required for normal embryonic development and can compensate for the lack of APP or APLP1.

APP, APLP1 and APLP2 undergo shedding in their ectodomain by proteases referred to as α- and β-secretases, followed by γ-secretase-mediated intramembrane proteolysis. The cleavage of APP is intensively studied, as APP is the precursor for the amyloid β peptide (Aβ), which has a key role in Alzheimer's disease (AD) pathogenesis [11]. APP cleavage by β- and γ-secretase generates Aβ, whereas cleavage by α-secretase prevents Aβ generation [12], [13]. APLP1 and APLP2 do not contain an Aβ domain and are not assumed to contribute to AD.

Despite the crucial function of APLP2 as described above, much less is known about the proteolytic cleavage of APLP2 compared to APP. Similar to APP, the type I membrane protein APLP2 undergoes shedding by the β-secretase BACE1 (β-site APP cleaving enzyme), which results in secretion of the N-terminal APLP2 ectodomain and in generation of the corresponding membrane-bound C-terminal fragments (CTFs) [14], [15], [16], [17], [18]. APLP2 also undergoes shedding by α-secretase. Although the name α-secretase was initially coined for the metalloprotease cleaving APP, α-secretase is now used more widely to indicate the shedding of membrane proteins by a metalloprotease, typically of the ADAM (A disintegrin and metalloprotease) family. As expected for a cleavage by ADAM proteases, α-secretase cleavage of APLP2 occurs constitutively and is reduced by the metalloprotease inhibitors TAPI-2 or GM6001 [14], [15]. Moreover, APLP2 α-secretase cleavage can be stimulated above its constitutive level by the phorbol ester PMA [14]. The constitutive α-secretase cleavage of APLP2 seems to be mediated by ADAM10 and ADAM17 (also known as TACE - TNFα converting enzyme). Overexpression of ADAM10 or ADAM17 in kidney cells and of ADAM10 in mouse brain increases APLP2 shedding [14], [19]. Conversely, the metalloprotease inhibitor GI254023X reduces APLP2 shedding down to 30%. Because this inhibitor is 100-fold more potent in inhibiting recombinant ADAM10 than ADAM17, it was concluded that ADAM10 may be the primary α-secretase for APLP2 [14]. However, overexpression of a dominant-negative ADAM10 reduced APLP2 shedding only by 40% [14], raising the possibility that ADAM17 is also involved in constitutive APLP2 shedding. In fact, ADAM17, but not ADAM10, is required for APLP2 shedding in SH-SY5Y cells stimulated with insulin-like growth factor-1 [20]. So far, the constitutive APLP2 shedding has not yet been analyzed in primary neurons. Taken together, it remains unclear whether only ADAM10 or also ADAM17 contributes to the constitutive α-secretase cleavage of APLP2. A similar situation was observed for APP, where ADAM10 and 17 as well as other metalloproteases were discussed as the constitutive APP α-secretases. Recently, we demonstrated that only ADAM10, but not ADAM17, mediates the constitutive APP α-secretase cleavage in different cell lines and in primary murine neurons [21].

Ectodomain shedding can produce soluble protein fragments with biological activity, for example growth factors and cytokines [3], [4]. Likewise, it is clear that the soluble APP ectodomain can be biologically active. For example, the APP ectodomain released through β-secretase cleavage (APPsβ) binds and activates the death receptor DR6, acting in a proapoptotic manner [22]. Additionally, it is able to induce expression of transthyretin and Klotho [23]. In contrast, the α-secretase-cleaved APP (APPsα) has neurotrophic and neuroprotective properties [24], [25], [26] and is able to rescue the mild phenotype of APP single knock-out mice [27]. Together, this demonstrates that APPsα and APPsβ have different biological functions, although APPsα is only 16 amino acids longer than APPsβ [28]. Thus, it is essential to know the cleavage sites and the identity of the contributing protease activities in order to study the physiological functions of the APP cleavage products in depth. Compared to APP much less is known about the proteolytic cleavage of APLP2 and its functional consequence. The α-, β- and γ-cleavage sites for APLP2 have not yet been determined [5]. Thus, it remains unknown whether – similar to APP – α- and β-secretase produce APLP2 ectodomains of different length and diverse functions.

Here we demonstrate that the constitutive α-secretase cleavage of APLP2 in HEK293 and in neuroblastoma SH-SY5Y cells is mediated by ADAM10, but not by ADAM17. Furthermore, knock-down of ADAM10 completely abolished α-secretase cleavage of APLP2 in primary murine neurons, demonstrating that ADAM10 is the constitutive α-secretase for APLP2. Moreover, we determined the cleavage sites of α-, β- and γ-secretase in the APLP2 sequence.

## Results

### APLP2 is processed by an α-secretase

**Figure 1:**
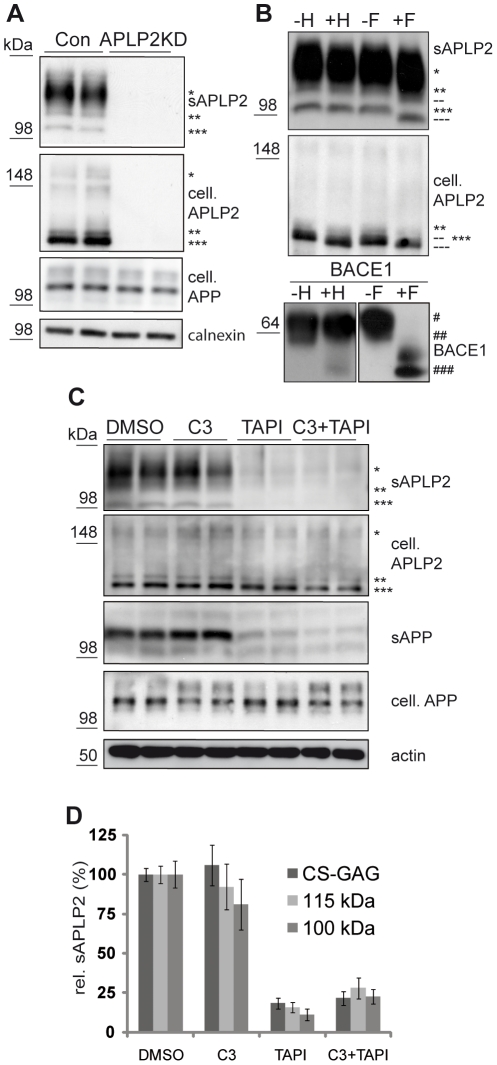
Analysis of APLP2 shedding using protease inhibitors. (**A**) Human neuroblastoma SH-SY5Y cells were transiently transfected with an siRNA pool against APLP2 or with a control pool. Endogenous APLP2 was detected with the N-terminally binding 2D11 antibody. Bands were absent upon knock-down of APLP2 (APLP2KD), demonstrating the specificity of the antibody. Soluble APLP2 (sAPLP2) was detected in the conditioned medium, cellular full-length APLP2 (cell. APLP2), cellular full-length APP (cell. APP) and calnexin as a loading control in the cell lysate. Three forms of APLP2 were detected: CS-GAG modified (*) and two non-CS-GAG modified species (** and ***) with molecular weights of around 115 and 100 kDa respectively. (**B**) Deglycosylation of APLP2 in conditioned medium and cell lysate of SH-SY5Y cells using endoglycosidase H (H) and N-glycosidase F (F). Deglycosylated forms of APLP2 are indicated (-- and ---). As a control, deglycosylation was performed for BACE1 in the cell lysate of BACE1 overexpressing HEK293 cells. Mature (#), immature (##) and deglycosylated (###) BACE1 is detectable. (**C**) Representative blots of treatment of SH-SY5Y cells with C3 (1 µM), TAPI-1 (50 µM) or C3+TAPI-1. Upon C3 treatment no significant reduction of sAPLP2 levels was observed. Upon TAPI-1 and C3+TAPI-1 treatment sAPLP2 levels were strongly reduced while no changes in APLP2 levels in the cell lysate were observed. As a control, soluble APP (sAPP) levels were clearly reduced upon TAPI-1 and C3+TAPI-1 treatment. (**D**) Quantification of experiments in C (mean +/− SEM). C3 treatment did not lead to a significant reduction in sAPLP2 levels while TAPI-1 as well as C3+TAPI-1 treatment led to a significant reduction in sAPLP2 levels (p<0.001 for all three species, n = 6).

Human neuroblastoma SH-SY5Y cells shed three different soluble species (sAPLP2) of the endogenous APLP2 (Fig. 1A). As reported previously [14], [29], [30], the broad band between 120 and 160 kDa (***) corresponds to sAPLP2 carrying chondroitin sulfate glycosamino glycans (CS-GAG) at serine 614 of the 751 amino acids long APLP2 isoform 2. Due to heterogeneity in the amount of glycosylation, this form is detected as a broad band. The two sharp bands at apparent molecular weights of 115 and 100 kDa are assumed to be non-CS-GAG modified, mature sAPLP2 (**) and a truncated form of non-CS-GAG modified, mature sAPLP2 (***), respectively. sAPLP2 species are complex glycosylated as shown by their resistance to endoglycosidase H treatment. In the cell lysate full-length APLP2 was detected as two immature bands (marked ** and ***, Fig. 1A), which were endoglycosidase H sensitive (Fig. 1B), and as the CS-GAG modified APLP2 (*; Fig. 1A). All protein bands are specific for APLP2, because they were absent upon RNAi-mediated knock-down of APLP2 (Fig. 1A). As a positive control for the deglycosylation assay, deglycosylation was performed for the β-secretase BACE1. In agreement with previous studies [31], [32], [33], the mature form (#) was sensitive to N-glycosidase F, but not to endoglycosidase H, whereas the immature form (##) was sensitive to both glycosidases.

Next, we determined to which extent APLP2 shedding occurs by α- and β-secretase in SH-SY5Y cells. Treatment with the broad spectrum metalloprotease inhibitor TAPI-1, which also inhibits ADAM proteases, reduced APLP2 shedding down to less than 20% in SH-SY5Y cells (Fig. 1C and quantification in Fig. 1D), demonstrating that APLP2 shedding is mediated to a large extent by α-secretase, in agreement with previous publications [14], [15]. As a control, TAPI-1 also inhibited the shedding of APP (Fig. 1C). The inhibitor C3, which is specific for the β-secretase BACE1 [34], did not lead to a significant reduction of total APLP2 shedding, revealing that β-secretase cleavage only contributes to a minor extent to total APLP2 shedding. This was further confirmed by the combined use of TAPI-1 and C3, which did not decrease total APLP2 shedding further than the use of TAPI-1 alone (Fig. 1C and D). The remaining small amount of APLP2 shedding may result from an as yet unknown protease different from α- and β-secretase. Alternatively, TAPI-1 may not be able to completely block α-secretase cleavage of APLP2.

### Constitutive α-secretase cleavage of APLP2 is mediated by ADAM10 in HEK293 and SH-SY5Y cells

**Figure 2:**
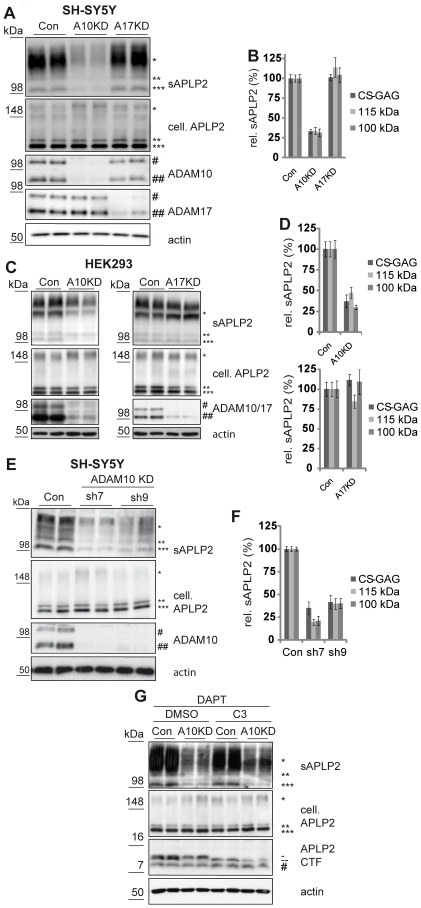
Transient and stable knock-down of ADAM10 suppresses APLP2 shedding. (**A**) SH-SY5Y cells were transfected with siRNA pools against the proteases ADAM10 (A10KD) or ADAM17 (A17KD) or with control siRNA (Con). Both proteases were detected in membrane preparations. The mature active form is indicated with ##, the immature form with #. Actin and full-length APLP2 levels (cell. APLP2) were detected in the cell lysate. Conditioned media were analyzed for total secreted APLP2 (sAPLP2). All three species of sAPLP2 (* CS-GAG modified, ** 115 kDa, *** 100 kDa) were strongly decreased upon knock-down of ADAM10, but not of ADAM17. (**B**) Quantification of experiments in A (mean +/− SEM). ADAM10 knock-down significantly reduced sAPLP2 (p<0.001 for all three species, n = 6), while ADAM17 knock-down did not lead to any significant changes in sAPLP2 levels. (**C**) Knock-down of ADAM10 and ADAM17 in HEK293 cells, carried out as in A. (**D**) Quantification of experiments in C (mean +/− SEM). ADAM10 knock-down significantly reduced sAPLP2 (p<0.001 for CS-GAG modified and 100 kDa APLP2, p = 0.001 for 115 kDa APLP2, n = 6). (**E**) SH-SY5Y cells with stable shRNA-mediated knock-down of ADAM10 were used. shRNAs sh7 and sh9 are targeting two different regions of ADAM10. As control, a stable SH-SY5Y cell line expressing a non-targeting shRNA was used (Con). sAPLP2 levels were clearly reduced upon ADAM10 knock-down. (**F**) Quantification of experiments in E (mean +/− SEM). Both shRNAs significantly reduced sAPLP2 (p<0.001 for all three species, n = 6). (**G**) SH-SY5Y cells were transiently transfected with a siRNA pool against ADAM10 (A10KD) or control siRNA (Con). Cells were treated with DAPT (1 µM). Additionally C3 (1 µM) or DMSO (as a solvent control) was applied. sAPLP2 levels were clearly reduced upon ADAM10 knock-down while cellular APLP2 levels (cell. APLP2) remained unchanged. Two forms of APLP2 C-terminal fragments (CTFs) were detected at around 10 kDa in the cell lysate (-,--). ADAM10 knock-down led to the elimination of the lower molecular weight APLP2 CTF (--) while C3-treatment abolished the higher molecular weight species (-).

To determine whether constitutive α-secretase cleavage of APLP2 is mediated by ADAM10 or ADAM17 or both, a transient knock-down of both proteases was performed in SH-SY5Y cells (Fig. 2A and quantification in Fig. 2B). The siRNA pools efficiently reduced the protein levels of ADAM10 and ADAM17 (Fig. 2A). Knock-down of ADAM17 did not affect APLP2 shedding, ruling out the possibility that ADAM17 is the constitutive APLP2 α-secretase. In contrast knock-down of ADAM10 inhibited APLP2 shedding by ∼70%, which is similar to the reduction of APLP2 shedding obtained with the metalloprotease inhibitor TAPI-1 (Fig. 1D). The inhibitor was slightly more potent than the siRNA in reducing APLP2 shedding, most likely due to the fact that the siRNA-mediated knock-down is not able to completely suppress ADAM10 protein levels. In a previous study we found 10–15% of ADAM10 protein remaining in the siRNA treated cells [21]. The shedding of all three sAPLP2 species was reduced to a similar extent (Fig. 2A and quantification in 2B). To rule out the possibility that the results obtained are cell line-specific, the same experiment was carried out in human embryonic kidney 293 cells (HEK293). Very similar to the SH-SY5Y cells, ADAM10, but not ADAM17, was essential for α-secretase cleavage of APLP2 (Fig. 2C and quantification in 2D).

To further validate this finding, SH-SY5Y cells with a stable knock-down of ADAM10 were used [21]. Similar to the transient ADAM10 knock-down, both shRNA sequences, which efficiently reduce ADAM10 protein levels [21], inhibited APLP2 shedding by 60–80% (Fig. 2E and quantification in 2F).

The α-secretase ADAM10 generates the soluble APLP2 ectodomain and additionally the membrane-bound C-terminal fragments (CTFs) of APLP2, which should be reduced in ADAM10 knock-down cells. As the CTFs are rapidly further cleaved by γ-secretase, we stabilized them with the γ-secretase inhibitor DAPT [35] in wild-type SH-SY5Y cells or cells with a knock-down of ADAM10. DAPT treatment led to an accumulation of the CTFs (Fig. 2G), which were not visible in the absence of DAPT or upon knock-down of APLP2 (not shown). Three CTF bands were detected. Two have an apparent molecular weight of approximately 10 kDa (-, --), in agreement with previous publications [14], [17], [18]. One additional study detected six different CTFs, which may be specific for the overexpression of a C-terminally tagged APLP2 construct used in that study [15]. The third band that we observed is at approx. 8 kDa (#, Fig. 2G). The lower of the two bands at 10 kDa was absent in the ADAM10 knock-down cells, demonstrating that it is the ADAM10-generated CTF. The upper band was absent upon treatment with the β-secretase inhibitor C3 (Fig. 2G), showing that it is produced by the β-secretase BACE1, which is in agreement with previous studies analyzing the CTFs generated upon BACE1 overexpression or knock-out [17], [18]. The intensity of the CTFs may suggest a relatively large contribution of BACE1 to total APLP2 cleavage, whereas the shedding analysis (Fig. 1B) revealed only a minor contribution of BACE1 to APLP2 shedding. This difference is likely due to the fact, that a γ-secretase inhibitor was used for detecting the endogenous APLP2 CTFs, such that also the β-secretase generated CTF accumulates, even if it is only generated at much lower levels compared to the α-secretase generated CTF. Levels of the 8 kDa CTF were not significantly affected by ADAM10 knock-down or C3 treatment (t-test significance value p>0.1; n = 6), suggesting that it is generated by an additional protease unrelated to ADAM10 and BACE1. Because this fragment was not described in previous studies, it may only be seen upon γ-secretase inhibition.

From the above experiments we conclude that ADAM10, but not ADAM17, is required for the constitutive APLP2 α-secretase cleavage in SH-SY5Y and HEK293 cells.

### APLP2 shedding in primary neurons

**Figure 3:**
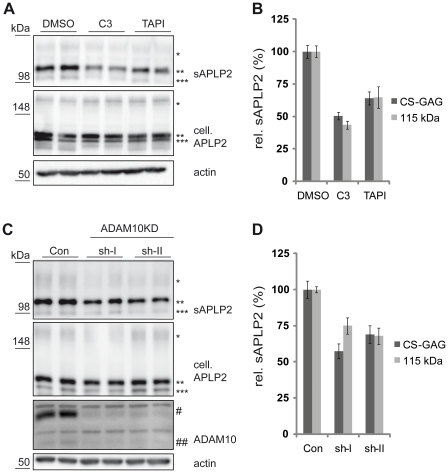
APLP2 processing in primary cortical neurons. (**A**) E16 primary cortical neurons were treated with C3 (1 µM), TAPI-1 (50 µM) or DMSO as a solvent control. sAPLP2 (* CS-GAG-modified, ** 115 kDa, *** 100 kDa) was detected in the conditioned medium of these cells. Full-length cellular APLP2 (cell. APLP2) and actin were detected in the cell lysate. A clear reduction of sAPLP2 levels was observable upon C3 as well as TAPI-1 treatment. (**B**) Quantification of experiments in A (mean +/− SEM). C3 and TAPI-1 significantly reduced sAPLP2 (C3: p<0.001 for both detected species, TAPI-1: p<0.001 for CS-GAG modified APLP2, p = 0.004 for mature APLP2; n = 6). (**C**) E16 primary cortical neurons with lentiviral knock-down of ADAM10 were analyzed. shRNAs (sh-I and sh-II) targeting different regions of ADAM10 and a non-targeting shRNA control (Con) were used. A clear reduction of ADAM10 (# immature, ## mature) levels was observed in the cell lysate and of sAPLP2 in the conditioned medium. (**D**) Quantification of experiments in C (mean +/− SEM). Both shRNAs significantly reduced sAPLP2 (p<0.01 for both detected species for both shRNAs, n = 6).

Next, we analyzed whether ADAM10 is also required for APLP2 α-secretase shedding in primary neurons. Embryonic neurons were prepared at E16. First, we used pharmacological inhibitors of α- and β-secretase to evaluate their contribution to the total amount of APLP2 shedding in neurons. The specific β-secretase inhibitor C3 [34] reduced endogenous sAPLP2 levels by about 50%, whereas the metalloprotease inhibitor TAPI-1 reduced it by about 35% (Fig. 3A and quantification in 3B). Thus, compared to HEK293 cells, APLP2 shedding in neurons is mediated to a larger extent by the β-secretase BACE1. This is similar to APP and is in agreement with the finding that BACE1 is expressed at higher levels in neurons than in cell lines [21], [36], [37]. Due to alternative splicing of APLP2, which destroys the CS-GAG attachment site, neurons have less of the CS-GAG-modified APLP2 compared to HEK293 or SH-SY5Y cells (Fig. 2A, B) [38], [39]. Next, ADAM10 was efficiently knocked-down in the primary neurons (Fig. 3C) using lentiviruses expressing two distinct and validated shRNAs against ADAM10 [21], [40]. sAPLP2 levels were reduced by about 30% (Fig. 3C and quantification in 3D). This is similar to the metalloprotease inhibitor TAPI-1 (Fig. 3A) and demonstrates that the constitutive α-secretase cleavage of APLP2 in neurons is mediated by ADAM10.

### APLP2 α-secretase cleavage occurs at the Arg670-Val671 peptide bond

**Figure 4:**
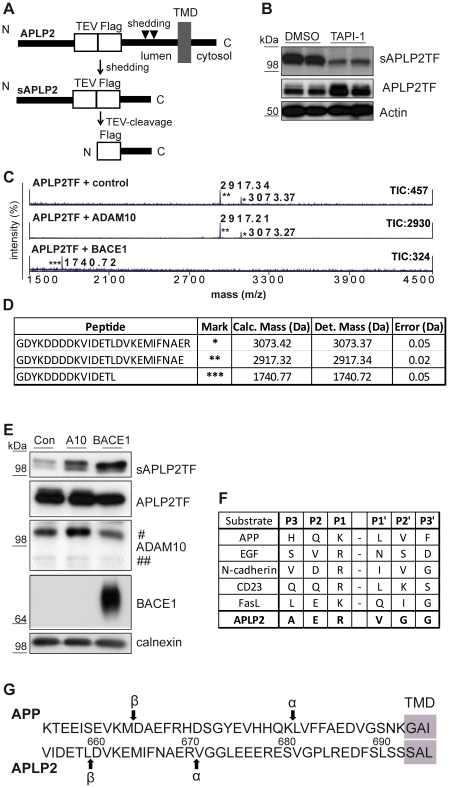
Mass spectrometry-based determination of APLP2 α- and β-shedding sites. (**A**) Scheme of experimental procedure. A TEV-protease cleavage site followed by a FLAG-tag was introduced into APLP2 isoform 763 starting after amino acid M653 (APLP2TF). The TEV-FLAG site is positioned 39 amino acids N-terminally of the assumed start of the transmembrane domain (TMD). Shedding yields sAPLP2TF, which was immunoprecipitated and digested with TEV-protease, leading to a small peptide harboring the N-terminal FLAG-tag as well as the C-terminal cleavage site resulting from the shedding. This peptide was analyzed in a mass spectrometer. (**B**) HEK293 cells were transiently transfected with the APLP2TF construct and either treated with TAPI-1 (50 µM) or DMSO as a solvent control. Upon TAPI-1 treatment secreted sAPLP2TF levels were clearly decreased in the conditioned medium of the cells. An accumulation of APLP2TF levels was observed upon TAPI-1 treatment in the cell lysate. Actin was analyzed in the cell lysate as a loading control. (**C**) Peptides produced according to the scheme in A were obtained from HEK293 cells transiently overexpressing APLP2TF and either luciferase as a control, ADAM10 or BACE1. Mass spectrometric analysis of the peptides revealed two peaks for control and ADAM10 overexpression (*,**) while upon BACE1 overexpression only one peak with a lower m/z ratio was observed. Total Ion Counts (TIC) and centroid peak masses of the first isotopic peak are given for each isotopic peak cluster. All peaks are revealed from singly-charged peptides. (**D**) Determined (Det.) masses of the peaks in C (*,**,***) were compared to calculated (calc.) masses. For each mass, a corresponding peptide could be computed with less than 0.5 Da error. (**E**) Western Blot analysis of the samples used in C. sAPLP2TF was detected in the conditioned media, full-length APLP2 (APLP2TF), ADAM10 (A10, # immature and ## mature), BACE1 and calnexin as a loading control were detected in the cell lysates. A clear increase in sAPLP2TF levels was observed upon ADAM10 and BACE1 overexpression. sAPLP2TF has a slightly reduced apparent molecular weight upon BACE1 overexpression if compared to control (Con). (**F**) Summary of known ADAM10 cleavage sites in different substrates aligned with the newly detected ADAM10 cleavage site for APLP2 [41]. (**G**) Schematic comparison of the ectodomain shedding sites of APP and APLP2. The APP α-cleavage occurs 12 amino acids N-terminally of the transmembrane domain (TMD), for APLP2 it occurs 22 amino acids N-terminally of the TMD. For both proteins β-cleavage occurs N-terminally of an aspartate.

The cleavage sites of APLP2 by either ADAM10 or BACE1 have not yet been identified. To determine them, mass spectrometry was used. The intensive glycosylation of APLP2 would lead to a broadening of the peaks in the mass spectrometric analysis, making identification of specific cleavage sites difficult. Thus, similar to a previous study analyzing APP cleavage [21], two short peptide tags were included into the APLP2 ectodomain C-terminally of the glycosylation sites at a distance of 39 amino acids from the suggested transmembrane domain (Fig. 4A). One of the two peptide sequences encodes a tobacco etch virus (TEV) protease cleavage site, the other one encodes a FLAG tag. This mutant APLP2-TEV-FLAG construct (APLP2TF) was transiently expressed in HEK293 cells. The metalloprotease inhibitor TAPI-1 strongly inhibited shedding of APLP2TF (Fig. 4B), demonstrating that APLP2TF was processed like the wild-type, endogenous APLP2 (Fig. 1B).

For this study the longest APLP2 isoform (763 amino acids) was used, which lacks the CS-GAG modification. The secreted form of APLP2-TF was immunoprecipitated from the conditioned medium with an anti-FLAG antibody and then digested in vitro with TEV protease. This leads to the removal of the glycosylated part of the APLP2 ectodomain, resulting in ∼3 kDa peptides having the FLAG tag at their new N-terminus and C-terminally ending at the peptide bond, where APLP2 is shed by the secretases (Fig. 4A). Mass spectrometric measurements yielded two peptide peaks at 2917.34 Da and at 3073.37 Da, which correspond to the peptides having glutamate 669 and arginine 670 respectively as their C-terminal amino acid (Fig. 4C and D). Overexpression of ADAM10 strongly increased APLP2TF shedding (Fig. 4E). The same two peptide peaks were observed under control conditions, but with a much higher intensity (TIC, total ion count, Fig. 4C), indicating that both peptides directly derive from ADAM10-mediated cleavage. Given that ADAM10 preferentially cleaves after arginine or lysine [41], we assume that the primary ADAM10 cleavage site in APLP2 is after arginine 670 and then followed by an as yet unidentified carboxypeptidase cleavage, similar to what is known for APP [21], [42]. This is in agreement with our observation that the ratio of the two peptides depends on the duration of the incubation of the supernatant on the cells, with longer incubations leading to a shift towards the shorter product (data not shown).

Overexpression of BACE1 increased APLP2TF shedding (Fig. 4E) and suppressed the ADAM10-generated peptide peaks and yielded a shorter peptide with 1740.72 Da (Fig. 4C), corresponding to leucine 659 as the C-terminal amino acid (Fig. 4D). The leucine is in good agreement with the substrate specificity of BACE1 known from in vitro experiments [43] and is also found in other BACE1 substrates, such as the Swedish variant APP and P-selectin glycoprotein ligand-1 [44] (Fig. 4G). The BACE1-generated peptide was not detected under control conditions. As the BACE1 cleavage makes up a very small proportion of total APLP2 shedding (see C3-inhibitor treatment in Fig. 1B), it is likely that this peptide was below the detection limit in our analysis.

### Heterogeneous γ-secretase cleavage occurs between alanine 694 and valine 700

**Figure 5:**
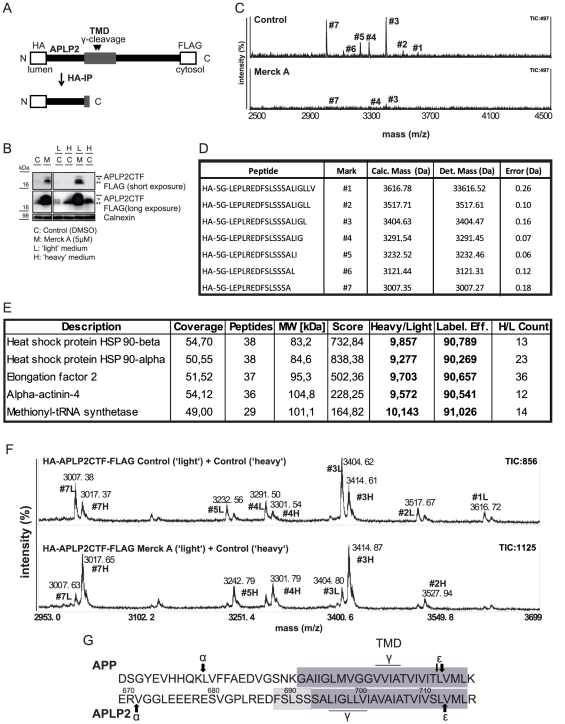
Mass spectrometry-based determination of APLP2 γ-cleavage site. (**A**) Scheme of experimental procedure: the HA-APLP2CTF-FLAG construct was stably expressed in HEK293 cells. The construct consists of an N-terminal HA-tag (HA), a linker region consisting of five glycines (5G), the APLP2CTF sequence (APLP2) and an C-terminal FLAG-tag (FLAG). Upon γ-secretase cleavage, peptides harboring the γ-secretase cleavage site at their C-terminus were liberated. Peptides were immunoprecipitated using HA-affinity agarose and subsequently analyzed in a MALDI-TOF mass spectrometer. (**B**) Western Blot to confirm the γ-secretase sensitivity of the construct. Cells stably expressing HA-APLP2CTF-FLAG were treated with (M) or without (C, control) the γ-secretase inhibitor Merck A. In the presence of Merck A the CTFs accumulated in the lysate. The same was observed for cells grown in ‘light’ (L) or ‘heavy’ (H) medium. CTFs were detected with anti-FLAG antibody. Calnexin levels were analyzed as a loading control. (**C**+**D**) Mass spectrometry-based identification of the γ-secretase cleavage sites. Under control conditions, seven peaks were detected with a maximal total ion count of 497. Upon Merck A treatment, the intensity (ion count) for the peaks was reduced significantly. Peaks are labeled with identifiers #1–#7 linking them to the respective peptides sequences in the table (D). All peptides were detected with a mass error of less than 0.3 Da. (**E**) Determination of the labeling efficiency for the SILAC experiment. To determine the labeling efficiency, proteins from the cell lysates of the ‘heavy’ labeled cells from (F) were separated on an SDS-PAGE gel. One band was cut out and tryptic in gel digestion was performed. Peptides resulting from this digestion were analyzed in an LTQ Orbitrap Velos mass spectrometer. Proteins were identified by database search, and the labeling efficiency (Label. Eff.) was determined from the ‘heavy’ to ‘light’ ratios (Heavy/Light) of the quantifiable peptides. Further, coverage of the proteins in % (Coverage), number of unique peptides identified (Peptides), molecular weight of respective protein (MW), SEQUEST score (Score) and number of unique peptides used for quantification (H/L Count) are given. (**F**) Analysis of γ-secretase-dependent CTF cleavage by quantitative mass spectrometry. Upper panel: Cells without Merck A (Control) grown either in ‘light’ or in ‘heavy’ medium. Supernatants were combined, peptides immunoprecipitated and analyzed in the MALDI-TOF mass spectrometer. Peaks resulting from the ‘light’ and the ‘heavy’ labeled peptides are clearly separated (+10 Da as expected for one heavy labeled arginine). Intensities of corresponding ‘heavy’ and ‘light’ peak clusters are not identical, as the ‘heavy’ clusters are either shifted (+10 Da) or supershifted (+16 Da) due to additional incorporation of a ‘heavy’ proline resulting from arginine- to-proline conversion. Lower panel: upon treatment of the ‘light’ labeled cells with Merck A and the ‘heavy’ labeled cells with DMSO as a solvent control, the ratio between the intensity of the ‘light’ labeled peptides and the ‘heavy’ labeled peptides is significantly reduced if compared to the control experiment. This demonstrates that the identified peptide peaks are generated by γ-secretase. (**G**) Comparison of the position of the ε- and γ-secretase cleavage sites of APP and APLP2 in an alignment of the juxtamembrane and the transmembrane region of these two proteins. The dark boxes mark the predicted transmembrane domains (TMD), while the light box indicates amino acids which could potentially still be part of the transmembrane domain, as they do not harbor charges.

After ectodomain shedding APLP2 undergoes intramembrane proteolysis by γ-secretase [15]. While the so-called ε-cleavage site at the C-terminal end of APLP2 has been mapped to occur after leucine 712 [45], the γ-cleavage sites remain unknown. For identification of the γ-secretase cleavage sites the APLP2 CTF was stably expressed in HEK293 cells. The CTF construct was composed of the N-terminal HA-tag, a short linker region, followed by the APLP2-CTF starting on proline 683. The construct further harbored a C-terminal FLAG-tag fused to the end of the CTF (Fig. 5A, HA-APLP2CTF-FLAG). As a control, the γ-secretase inhibitor Merck A [46] blocked γ-secretase cleavage of the CTF, as evidenced by increased CTF levels (Fig. 5B).

The secreted peptides resulting from γ-secretase cleavage were immunoprecipitated from the supernatant and analyzed by MALDI-TOF mass spectrometry. Seven peaks were identified in the range of 3000 to 3700 Da with the main peak showing a mass of 3404.47 Da (Fig. 5C). This implies that the main γ-secretase cleavage site is the peptide bond between leucine 698 and leucine 699 (Fig. 5D). Multiple γ-secretase cleavage sites are typically detected for γ-secretase substrates, including APP [47].

As a control, we verified that the identified peptides were generated in a γ-secretase-dependent manner. In fact, the γ-secretase inhibitor Merck A strongly reduced the seven mass peaks (Fig. 5C). Because mass spectrometry is only semi-quantitative, we further verified this finding by using a truly quantitative mass spectrometry technique, called SILAC (stable isotope labeling with amino acids in cell culture) [48]. In this approach cells from two different experimental conditions, such as with and without Merck A, are grown in the presence of isotopically light (with Merck A) or heavy (without Merck A) amino acids arginine and lysine. The combined supernatants are then analyzed by mass spectrometry. To demonstrate the method, the stably HA-APLP2CTF-FLAG expressing HEK293 cells were first grown without Merck A (control) in either light or heavy medium. Labeling efficiency was above 90% as determined by mass spectrometry based analysis of the cell lysate of the heavy labeled cells (Fig. 5E). Upon growth in the ‘heavy’ medium, the cells incorporated the heavy labeled amino acids into their proteins thereby increasing the molecular weight of the APLP2 CTF-derived peptides by +10 Da due to the single arginine found in every of the seven peptides (Fig. 5F, upper panel). Addition of Merck A to the ‘light’ labeled cells strongly reduced the ‘light’ peaks relative to the ‘heavy’ peaks from the solvent control (DMSO)-treated cells (Fig. 5F, lower panel), demonstrating that all seven peaks result from γ-secretase cleavage.

## Discussion

Ectodomain shedding regulates the activity of membrane proteins and is frequently the regulatory step for subsequent intramembrane proteolysis [2], [49]. Shedding is often mediated by ADAM-proteases, in particular by ADAM10 and ADAM17. Both proteases have overlapping specificities in vitro [41] and overexpression of either one can increase the shedding of some substrates, whereas under physiological conditions frequently only of the two proteases cleaves a given substrate. Here, we demonstrate that this is also the case for APLP2. We found that the constitutive α-secretase cleavage of the APP homolog APLP2 is mediated by ADAM10, but not by ADAM17. Also in neurons, where we investigated APLP2 shedding for the first time, ADAM10 was essential for constitutive APLP2 α-secretase cleavage. A similar situation was previously found for APP. Although ADAM10 and ADAM17 and even ADAM9 can cleave APP and stimulate its shedding under overexpression conditions [50], [51], [52], only ADAM10, but not ADAM9 or ADAM17, was recently shown to mediate the constitutive APP α-secretase shedding [21].

Our study reveals further similarities, but also differences between the constitutive shedding of the two homologs APP and APLP2. First, in cell lines, both proteins are predominantly cleaved by α-secretase and only to a low extent by β-secretase, whereas in primary neurons, which express higher levels of the β-secretase BACE1, APP and APLP2 are cleaved to at least 50% by β-secretase. Second, for both proteins the initial α-secretase cleavage appears to be followed by a carboxypeptidase cleavage removing the C-terminal, positively charged amino acid (Lys in APP, Arg in APLP2). While the functional relevance of this C-terminal truncation is not yet clear, it is interesting to note that the maturation of several prohormones also requires the removal of a C-terminal Lys or Arg by a carboxypeptidase [53]. Third, the α- and β-cleavage sites occur at similar peptide bonds, which are in agreement with the specificities of BACE1 and ADAM10 in vitro [41], [43]. Fourth, APP and APLP2 are cleaved at slightly different distances from the membrane. For APP, the α-cleavage occurs at a distance of 12 amino acids from the membrane. The β-cleavage is a further 16 amino acids away. For APLP2 the α-cleavage is at a distance of 22 amino acids from the membrane and the β-cleavage is a further 11 amino acids away (Fig. 4G). In contrast to APP, where a Lys is assumed to be the membrane-anchor and determines the beginning of the transmembrane domain, the boundaries of the transmembrane domain of APLP2 are less clear. The N-terminal boundary is assumed to start at serine 693 (Fig. 4G), but is preceded by several non-charged amino acids, which may also be part of the transmembrane domain. If that were the case, the distance of the α- and β-secretase cleavage sites in APLP2 would be even closer to the transmembrane domain and thus, even more similar to APP (Figs. 4G and 5G). The exact determination of the length of the APLP2 transmembrane domain would require biochemical approaches, as previously carried out for APP [54]. Although for APLP2 and APP the β-cleavage site is further away from the membrane than the α-secretase cleavage site, this is not the case for all membrane proteins undergoing shedding by an α- and a β-secretase. For example, for the cell adhesion protein P-selectin glycoprotein ligand-1, the cleavage site by β-secretase is closer to the membrane than the cleavage site by α-secretase [44].

Our finding that the constitutive α-secretase cleavage of APLP2 is mediated by ADAM10 is in agreement with previous reports showing that APLP2 shedding is reduced by a dominant-negative ADAM10 as well as by the metalloprotease inhibitor GI254023X [14], [19], which is more potent on ADAM10 than on ADAM17 [55]. However, another study reported that the knock-down of ADAM17, but not of ADAM10, reduced APLP2 shedding [20]. In that study, APLP2 shedding was not analyzed under constitutive conditions, but after stimulation with IGF-1. This demonstrates that the regulated APLP2 shedding – i.e. the increase above the constitutive shedding level – can be mediated by proteases other than the constitutively cleaving ADAM10. This is similar to APP, where the constitutive α-secretase cleavage is mediated by ADAM10, whereas the regulated α-secretase cleavage can be mediated by ADAM17, at least upon stimulation with the phorbol ester phorbol-12-myristate-13-acetate (PMA). Interestingly, the IGF-1-stimulated shedding, which requires ADAM17 for APLP2, requires ADAM10 for the APP cleavage [20]. This shows that α-secretase shedding of APP and APLP2 can be regulated in a different manner, despite the many similarities described above for the constitutive α-secretase cleavage. It will be interesting to see whether these differences also occur for other stimuli known to activate APP and APLP2 shedding.

In contrast to α- and β-secretase, we found that γ-secretase has multiple cleavage sites in the transmembrane domain of APLP2. This is also observed for other γ-secretase substrates, such as APP and Notch [47]. However, in contrast to APP the cleavage sites are not located in the middle of the predicted transmembrane domain, but are close to the N-terminal membrane boundary of APLP2 [15], [56]. In APP the main γ-cleavage site after amino acid 40 of the Aβ sequence is 12 amino acids away from both ends of the transmembrane domain. In contrast, the main cleavage site in APLP2 is only around six residues away from the N-terminal membrane boundary. A similar situation is found for the γ-secretase substrate Notch-1, where the S4 cleavage site is also only seven amino acids away from the N-terminal membrane boundary [57]. Nevertheless, it may well be possible that the transmembrane domain of APLP2 is in fact around five amino acids longer than predicted at the N-terminal side, as discussed above for the α- and β-secretase cleavage (Fig. 5G). In this case, the main γ-cleavage site in APLP2 would be 11 residues away from the N-terminal end of the transmembrane domain and thus – similar to APP – roughly be located in the middle of the transmembrane domain.

The determination of the α-, β- and γ-secretase cleavage sites is the basis for further developments in APLP2 research. It will allow the generation of cleavage site-specific antibodies, which distinguish between the sAPLP2 forms generated by α- or β-secretase. Such antibodies will not only be valuable tools for studying APLP2 cleavage by both proteases, but may also be useful for biomarker studies in AD. In this disease, an increase in protein levels of BACE1 and a reduction of α-secretase cleavage is observed [reviewed in 28], [58]. Potentially, the corresponding APLP2 cleavage fragments are changed in a similar way, which may be an additional approach to discriminate between AD patients and controls. This may be advantageous, as APLP2-derived peptides should not aggregate and not form deposits due to the lack of the Aβ domain. Thus, their levels should not be influenced by depositing peptides, similar to what was recently found for APLP1-derived Aβ-like peptides [59]. A second use of the cleavage sites of APLP2 may be the dissection of the function(s) of APLP2. Potentially, sAPLP2 generated by α- or β-secretase has different functions, similar to what is known for APP. α-secretase-cleaved APP acts in a neurotrophic and neuroprotective manner [24], [25], [26], whereas β-secretase cleaved APP may be proapoptotic [22].

Given that APPsα rescues the phenotype of APP single knock-out mice [27], it will be interesting to see whether expression of the α- or β-secretase-cleaved ectodomains of APP and APLP2 together are able to rescue the postnatal lethality in the APP/APLP2 double knock-out mice [9], [10]. Such studies will provide further insights not only into the function of APLP2, but also of APP and into the functional overlap or functional divergence between both homologs.

## Materials and Methods

### Reagents

The following antibodies were used: FLAG M2 (Sigma), HA.11 (Covance), ADAM10 (Calbiochem-422751), ADAM17 (Oncogene), APLP2 (Calbiochem-171617 and 171616) HRP-coupled anti-rabbit and anti-mouse (DAKO), HRP-coupled anti-rat (Santa Cruz), Calnexin (Stressgen), β-actin (Sigma), monoclonal antibody (mAb) 22C11 (anti-APP ectodomain) from Konrad Beyreuther. The following reagents were used: α-Cyano-4-hydroxy-cinnamic acid, anti-FLAG M2 and anti-HA affinity gel from Sigma-Aldrich, metalloprotease inhibitor TAPI-1, BACE inhibitor C3, γ-secretase inhibitor X (referred to as Merck A) [46] and Dodecyl maltoside (DDM) from Calbiochem; siRNA pools siGenome against ADAM10 and 17, APLP2 and corresponding controls from Dharmacon. Lipofectamine 2000, RNAimax and TEV protease from Invitrogen; endoglycosidase H and N-glycosidase F from Roche; Acetonitrile (ACN) and trifluoracetic acid (TFA) from Fluka. SILAC media were from Silantes.

### Cloning

The APLP2-TEV-FLAG (APLP2TF) construct was cloned by the introduction of a TEV-cleavage site (ENLYFQG) followed by a FLAG-tag (DYKDDDDK) into the juxtamembrane region of APLP2-763 subcloned into the pcDNA3.1 zeo (+) vector (Invitrogen). The short peptide motifs were introduced between the respective nucleotides corresponding to amino acids M653 and V654 of the APLP2-763 sequence. Plasmids peak12-ADAM10, peak12-BACE1 and peak12-luciferase have been described [44]. HA-APLP2CTF-FLAG construct was produced through subcloning of the APLP2CTF into the backbone of a pcDNA 3.1 zeo (+) vector containing the N-terminal HA-tag, the linker region consisting of five glycines and the C-terminal FLAG-tag, which was described before [60].

### Cell Culture, transfections, RNAi

HEK293-EBNA cells [21] were cultured in Dulbecco's modified Eagle medium (DMEM, Gibco) containing 10% fetal calf serum (FCS/Gibco) as described [61], [62]. SH-SY5Y cells were cultured in F12/DMEM (Lonza) supplemented with 15% FCS. SH-SY5Y cells with a lentivirus-induced stable knock-down of ADAM10 (shRNAs 7 and 9) were previously described [21]. Transient knockdown of ADAM10 and ADAM17 in HEK293E and SHSY5Y cells was performed as described [21]. In brief: Cells were transfected with either 10 nM (HEK293E) or 2 nM (SHSY5Y) of siGenome pool siRNA targeting ADAM10 or ADAM17 or the same amount of control siRNA (C2-pool) using RNAimax. After two days, transfection medium was replaced. After overnight incubation, conditioned medium and cell lysate (in 150 mM NaCl, 50 mM Tris pH 7.5, 1% Nonidet P-40) were collected. For detection of ADAM 10 and ADAM17, cell membranes were prepared as described previously [63]. Transient APLP2 knock-down was performed using the siGenome pool siRNA in SH-SY5Y cells (2 nM).

### Deglycosylation analysis

For deglycosylation analysis of APLP2, supernatants and cell lysates of SH-SY5Y cells were incubated either with or without 5 milliunits endoglycosidase H for 3 hours at 37°C or with or without 2.5 units of N-glysosidase F for 17 hours at 37°C. As a positive control, cell lysates from BACE1 overexpressing HEK293 cells were analyzed accordingly.

### Generation of lentiviruses and transduction of primary neurons

Primary neuronal cultures were obtained from the cerebral cortex of embryonic day (E16) C57/BL6 mouse embryos, incubated with 200 U of papain (Sigma Aldrich) (30 min at 34°C) and subsequently mechanically dissociated and transduced using lentiviral shRNA knock-down constructs as described [21], [64]. Breeding, maintenance and all experimental procedures on animals were approved and licensed by the Regierung von Oberbayern, Munich and were performed in accordance with German legislation and with the European Communities Council Directive (86/609/EEC).

### Inhibitor treatment

Inhibition of α-, β- and γ-secretase was performed using TAPI-1 (50 µM), C3 (1 µM) and DAPT (1 µM) respectively. Merck A (5 µM) was used as a further γ-secretase inhibitor. For SH-SY5Y or HEK293 cells, 1 or 2 days after plating, the supernatant was exchanged with supernatant containing the respective inhibitors and conditioned supernatants and cell lysates were collected 24 hours later. Inhibitor treatment of the primary neurons started at 7DIV and was performed for 24 hours.

### Determination of shedding sites

Determination of the shedding sites of APLP2 was performed as described for APP [21]. From the supernatant of HEK293 cells overexpressing pcDNA3.1/APLP2TF the shed ectodomain of APLP2TF was purified using anti-FLAG M2 agarose. The purified ectodomain was cleaved using TEV protease. The short peptide produced was again purified via anti-FLAG M2 agarose and subsequently analyzed in a Voyager-DE STR mass spectrometer (Applied Biosystems).

### Determination of γ-secretase cleavage sites

Peptides were purified from the supernatant from HEK293 cells stably overexpressing HA-APLP2CTF-FLAG applying anti-HA agarose and analyzed in a Voyager-DE STR mass spectrometer (Applied Biosystems). For γ-secretase inhibition supernatant from cells was removed and cells were treated with Merck A (5 µM) for 24 h prior to supernatant collection and subsequent analysis.

### SILAC experiment

HEK293 cells stably overexpressing HA-APLP2CTF-FLAG were grown either in ‘light’ (12C6, 14N2 Lys; 12C6 14N4 Arg) or in ‘heavy’ medium (13C6, 15N2 Lys; 13C6 15N4 Arg) for at least six cell doublings before the actual experiment. For this, identical numbers of cells were plated in ‘heavy’ or ‘light’ medium. After overnight incubation the enriched medium was collected and equal quantities from both ‘heavy’ and ‘light’ cell supernatant was combined prior to immunoprecipitation with the anti-HA agarose. Cell lysates were produced for subsequent determination of the labeling efficiency.

### Determination of labeling efficiency

For determination of the labeling efficiency, proteins from the cell lysates of the cells grown in the ‘heavy’ medium were separated on an SDS-Page gel. A band of the Coomassie stained gel at an apparent mass of around 90 kDa was cut out and tryptic in gel digestion of the proteins was performed [65]. Subsequently, peptides were analyzed by liquid chromatography (EASY nLC II, Proxeon) coupled to tandem mass spectrometry (LTQ Orbitrap Velos, Thermo) using a TOP10 method. Data analysis was performed using the Proteome Discoverer 1.2 (Thermo) with the embedded SEQUEST algorithm for protein identification. The International Protein Index database for human (version 3.78) was used for the database search with carbamidomethylation of cysteine as a static and oxidation of methionine as a dynamic modification. Quantification was performed using the ‘precursor ions quantifier’ node of the Proteome Discoverer 1.2.

Labeling efficiency was further validated in a control experiment. Equal amounts of supernatant from untreated ‘light’ and untreated ‘heavy’ labeled HA-APLP2CTF-FLAG HEK293 cells were combined and subsequent immunoprecipitation was performed as described above. The area under the isotopic peak cluster of every ‘light’ peptide was compared to the area under the isotopic peak cluster of the corresponding ‘heavy’ peptide. For the ‘heavy’ peptide, the +10 Da shifted (one 13C6 15N4 Arg per peptide) and the +16 Da supershifted (one 13C6 15N4 Arg and one 13C5 15N1 Pro per peptide) istopic peak clusters were combined, as arginine-to-proline conversion played a significant role in our experiment [66]. Labeling efficiency was also found to be above 90% when determined by this method.
